# Predicting alcohol dependence from multi‐site brain structural measures

**DOI:** 10.1002/hbm.25248

**Published:** 2020-10-16

**Authors:** Sage Hahn, Scott Mackey, Janna Cousijn, John J. Foxe, Andreas Heinz, Robert Hester, Kent Hutchinson, Falk Kiefer, Ozlem Korucuoglu, Tristram Lett, Chiang‐Shan R. Li, Edythe London, Valentina Lorenzetti, Luijten Maartje, Reza Momenan, Catherine Orr, Martin Paulus, Lianne Schmaal, Rajita Sinha, Zsuzsika Sjoerds, Dan J. Stein, Elliot Stein, Ruth J. van Holst, Dick Veltman, Henrik Walter, Reinout W. Wiers, Murat Yucel, Paul M. Thompson, Patricia Conrod, Nicholas Allgaier, Hugh Garavan

**Affiliations:** ^1^ Department of Psychiatry University of Vermont College of Medicine Burlington Vermont USA; ^2^ Department of Psychology University of Amsterdam Amsterdam the Netherlands; ^3^ Department of Neuroscience & The Ernest J. Del Monte Institute for Neuroscience University of Rochester School of Medicine and Dentistry Rochester New York USA; ^4^ Department of Psychiatry and Psychotherapy Charité—Universitätsmedizin Berlin Berlin Germany; ^5^ Melbourne School of Psychological Sciences University of Melbourne Melbourne Australia; ^6^ Department of Psychology and Neuroscience University of Colorado Boulder Colorado USA; ^7^ Department of Addictive Behaviour and Addiction Medicine, Central Institute of Mental Health Heidelberg University Mannheim Germany; ^8^ Department of Psychiatry Washington University School of Medicine St. Louis Missouri USA; ^9^ Department of Psychiatry Yale University School of Medicine New Haven Connecticut USA; ^10^ David Geffen School of Medicine University of California at Los Angeles Los Angeles California USA; ^11^ Monash Institute of Cognitive and Clinical Neurosciences & School of Psychological Sciences Monash University Melbourne Australia; ^12^ School of Psychology, Faculty of Health Sciences Australian Catholic University Melbourne Australia; ^13^ Department of Psychological Sciences the University of Liverpool Liverpool UK; ^14^ Behavioural Science Institute Radboud University Nijmegen the Netherlands; ^15^ Clinical NeuroImaging Research Core, Division of Intramural Clinical and Biological Research National Institute on Alcohol Abuse and Alcoholism Bethesda Maryland USA; ^16^ VA San Diego Healthcare System and Department of Psychiatry University of California San Diego La Jolla California USA; ^17^ Laureate Institute for Brain Research Tulsa Oklahoma USA; ^18^ Orygen, The National Centre of Excellence in Youth Mental Health Parkville Australia; ^19^ Centre for Youth Mental Health The University of Melbourne Melbourne Australia; ^20^ Department of Neurology Max Planck Institute for Human Cognitive and Brain Sciences Leipzig Germany; ^21^ Institute of Psychology, Cognitive Psychology Unit & Leiden Institute for Brain and Cognition Leiden University Leiden the Netherlands; ^22^ SA MRC Unit on Risk & Resilience in Mental Disorders, Department of Psychiatry & Neuroscience Institute University of Cape Town Cape Town South Africa; ^23^ Neuroimaging Research Branch Intramural Research Program, National Institute on Drug Abuse Baltimore Maryland USA; ^24^ Department of Psychiatry, Amsterdam UMC, Location AMC University of Amsterdam Amsterdam the Netherlands; ^25^ Department of Psychiatry VU University Medical Center Amsterdam the Netherlands; ^26^ Melbourne Neuropsychiatry Centre, Department of Psychiatry The University of Melbourne and Melbourne Health Melbourne Australia; ^27^ Imaging Genetics Center, Stevens Institute for Neuroimaging & Informatics, Keck School of Medicine University of Southern California California USA; ^28^ Department of Psychiatry Université de Montreal, CHU Ste Justine Hospital Montreal Quebec Canada

**Keywords:** addiction, alcohol dependence, genetic algorithm, machine learning, multi‐site, prediction, structural MRI

## Abstract

To identify neuroimaging biomarkers of alcohol dependence (AD) from structural magnetic resonance imaging, it may be useful to develop classification models that are explicitly generalizable to unseen sites and populations. This problem was explored in a mega‐analysis of previously published datasets from 2,034 AD and comparison participants spanning 27 sites curated by the ENIGMA Addiction Working Group. Data were grouped into a training set used for internal validation including 1,652 participants (692 AD, 24 sites), and a test set used for external validation with 382 participants (146 AD, 3 sites). An exploratory data analysis was first conducted, followed by an evolutionary search based feature selection to site generalizable and high performing subsets of brain measurements. Exploratory data analysis revealed that inclusion of case‐ and control‐only sites led to the inadvertent learning of site‐effects. Cross validation methods that do not properly account for site can drastically overestimate results. Evolutionary‐based feature selection leveraging leave‐one‐site‐out cross‐validation, to combat unintentional learning, identified cortical thickness in the left superior frontal gyrus and right lateral orbitofrontal cortex, cortical surface area in the right transverse temporal gyrus, and left putamen volume as final features. Ridge regression restricted to these features yielded a test‐set area under the receiver operating characteristic curve of 0.768. These findings evaluate strategies for handling multi‐site data with varied underlying class distributions and identify potential biomarkers for individuals with current AD.

## Graphical abstract

To identify neuroimaging biomarkers of alcohol dependence (AD) from structural magnetic resonance imaging, we developed classifiers on data collected from multiple sites. Exploratory data analysis revealed that inclusion of case‐ and control‐only sites led to the inadvertent learning of site‐effects. Evolutionary‐based feature selection leveraging leave‐one‐site‐out cross‐validation, to combat unintentional learning, yielded a test‐set area under the receiver operating characteristic curve of 0.768.

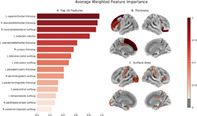

## 1 INTRODUCTION

While the evidence associating alcohol dependence (AD) with structural brain differences is strong (Ewing, Sakhardande, & Blakemore, 2014; Fein et al., 2002; Yang et al., 2016), there is considerable merit in establishing robust and generalizable neuroimaging‐based AD biomarkers (Mackey et al., 2019; Yip, Kiluk, & Scheinost, 2020). These biomarkers would have objective utility for diagnosis and may ultimately help in identifying youth at risk for AD and for tracking recovery and treatment efficacy in abstinence, including relapse potential. While these types of clinical applications have not yet been realized with neuroimaging, current diagnostic practices are far from perfect: The inter‐observer reliability of AD, as diagnosed by the DSM‐IV, was calculated with Cohen's kappa as 0.66 (0.54, 0.77, *n* = 171; Pierucci‐Lagha et al., 2007). More immediately, neurobiological markers of AD can give clues to potential etiological mechanisms.

Here, we apply a supervised learning approach, in which a function is trained to map brain structural measures to AD diagnosis, and then evaluated on unseen data. Prior approaches to developing machine learning classifiers for AD include a similar binary machine learning classification approach discriminating between AD and substance naive controls (Guggenmos et al., 2018). Their analysis made use of 296 participants, case and control, and reported a leave‐one‐out cross‐validated (CV) balanced accuracy of 74%. A further example of recent work includes that by Adeli et al. on distinguishing AD from controls (among other phenotypes), on a larger sample of 421, yielding a balanced accuracy across 10‐fold CV of 70.1% (Adeli, 2019). In both examples, volumetric brain measures were extracted and used to train and evaluate proposed machine learning (ML) algorithms. The present study differs from prior work in both its sample size (*n* = 2,034) and complex case to control distribution across a large number of sites. Mackey et al. (2019) developed a support vector machine (SVM) classifier that obtained an average area under the receiver characteristic operator curve (AUC) of 0.76 on a subset of the training data presented within this work. Our present study expands on this previous work by exploring new classification methods and additional samples with a focus on how to optimize cross‐validation consistent with generalization to new unseen sites. It is worth noting that the results from this previous work are not intended to be directly compared with the current work as the previous data were residualized (against pertinent factors including site) and only results from a split‐half analysis were computed (wherein each fold, by design, included participants from each site).

An important consideration for any large multi‐site neuroimaging study, particularly relevant in developing classifiers, is properly handling data from multiple sites (Pearlson, 2009). More generally and within the broader field of ML, the task of creating “fair” or otherwise unbiased classifiers has received a great deal of attention (Noriega‐Campero, Bakker, Garcia‐Bulle, & Pentland, 2019). We argue that in order for a classifier or biomarker to have utility, it must explicitly generalize to new data, possibly from a different scanner or country. Further, any information gleaned from a classifier that fails to generalize to new data is unlikely to represent the actual effect of interest. In our study, the imbalance between numbers of cases and controls across different sites is a significant challenge, as unrelated, coincidental scanner or site effects may easily be exploited by multivariate classifiers, leading to spurious or misleading results. We show that when datasets include sites containing only cases or only controls this can be a serious problem.

A related consideration is how one should interpret the neurobiological significance of features that contribute most to a successful classifier. We propose a multi‐objective genetic algorithm (GA) based feature selection search to both isolate meaningful brain measures and tackle the complexities of handling differing class distributions across sites. GA are considered a subset of evolutionary search optimization algorithms. A sizable body of research has been conducted into the usage of multi‐objective genetic algorithms, introducing a number of effective and general techniques to navigate high dimensional search spaces, including, various optimization and mutation strategies. (Coello, Lamont, & Veldhuizen, 2007; Deb, Pratap, Agarwal, & Meyarivan, 2002; Gen & Lin, 2007). Our proposed GA is designed to select a set of features both useful for predicting AD and generalizable to new sites. By selecting not just predictable, but explicitly generalizable and predictable features, we hope to identify features with true neurobiological relevance. We draw motivation from a large body of existing work that has successfully applied GAs to feature selection in varied machine learning contexts (Dong, Li, Ding, & Sun, 2018; Yang & Honavar, 1998).

**FIGURE 1:**
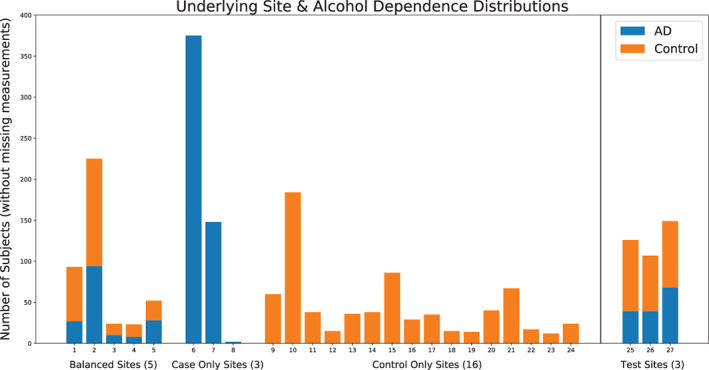
The distribution of both training (Sites 1–24) and testing (25–27) datasets is shown, and further broken down by AD to case ratio per site, as well as split by category (e.g., balanced vs. control‐only)

This study represents a continuation of work by Mackey et al. (2019) and the Enhancing Neuro‐Imaging Genetics through Meta‐Analysis (ENIGMA) Addiction Working Group (http://enigmaaddiction.com), in which neuroimaging data were collected and pooled across multiple laboratories to investigate dependence on multiple substances. Here, we focus on a more exhaustive exploration of machine learning to distinguish AD from nondependent individuals, spanning 27 different sites. Notably, individual sites are highly imbalanced, with most sites containing only participants with AD or only controls (see Figure 1). Due to the unavoidable presence of site‐related scanner and demographic differences, ML classifiers can appear to distinguish participants with AD, but are actually exploiting site‐related effects. In this context, we evaluate how different cross‐validation (CV) strategies can either reveal or hide this phenomenon, in addition to how choices around which sites to include (e.g., removing control‐only sites) can impact estimates of performance. We then introduce a GA based feature selection strategy and show how it can be used to address the unique concerns present in complex multi‐site data with varied underlying class distributions. Finally, we present classification results for a left‐out testing set sourced from three unseen sites, as a measure of classifier generalizability.

## 2 METHODS

### 2.1 Dataset

Informed consent was obtained from all participants and data collection was performed in compliance with the Declaration of Helsinki. Individuals were excluded if they had a lifetime history of any neurological disease, a current DSM‐IV axis I diagnosis other than depressive and anxiety disorders, or any contraindication for MRI. A variety of diagnostic instruments were used to assess alcohol dependence (Mackey et al., 2019). See Supporting Information for more specific details on the included studies.

Participants' structural T1 weighted brain MRI scans were first analyzed using FreeSurfer 5.3 which automatically segments 7 bilateral subcortical regions of interest (ROIs) and parcellates the cortex into 34 bilateral ROIs according to the Desikan parcellation. In total, we employ 150 different measurements representing cortical mean thickness (*n* = 68) and surface area (*n* = 68) along with subcortical volume (*n* = 14; Dale, Fischl, & Sereno, 1999; Desikan et al., 2006).

Quality control of the FreeSurfer output including visual inspection of the ROI parcellations was performed at each site according to the ENIGMA protocols for multi‐site studies, available at http://enigma.ini.usc.edu/protocols/imaging-protocols/. In addition, a random sample from each site was examined at the University of Vermont to ensure consistent quality control across sites. All individuals with missing volumetric or surface ROIs were excluded from analyses.

In total, 2,034 participants from 27 different sites met all inclusion criteria. Further, data were separated into a training set (used in an exploratory data analysis and to train a final model) composed of 1,652 participants (692 with AD), from 24 sites with the remaining 382 participants (146 with AD) from three sites isolated as a test set (used as a final left‐aside test of generalizability). The testing set represents a collection of new data submitted to the consortium that was not included in the most recent working group publication (Mackey et al., 2019). Table 1 presents basic demographic information on training and test splits. Within the training set, three sites contained only cases, 14 sites included only controls, and five sites contained a balanced mix in the number of cases and controls. Figure 1 shows the distribution by site, broken down by AD versus control. A more detailed breakdown of the dataset by study and collection site is provided within the supplemental materials.

**TABLE 1:** Sex and age, across the full collected dataset from 27 sites as split further into training and withheld testing set, and by alcohol use disorder (AD) versus control

Split‐AD	Participants	Male (%)	Mean age (*SD*)
Train‐AD	692	423 (61)	33.36 ± 9.96
Train‐Control	960	554 (57)	28.54 ± 9.56
Test‐AD	146	79 (54)	44.72 ± 10.55
Test‐Control	236	99 (42)	42.33 ± 12.31

### 2.2 Exploratory data analysis

In this section, we describe an exploratory analysis investigating different choices of training data, classification algorithms, and cross‐validation strategy. This step serves as an initial validation to ensure that the classification model of interest is actually learning to distinguish AD versus control versus exploiting an unintended effect. Further, this step allows us to explore how different choices of classifier and data affect performance, as the ultimate goal is to build as predictive a classifier as possible. A final framework for training is determined from this exploration, and its implementation and evaluation are covered in the following sections.

**FIGURE 2:**
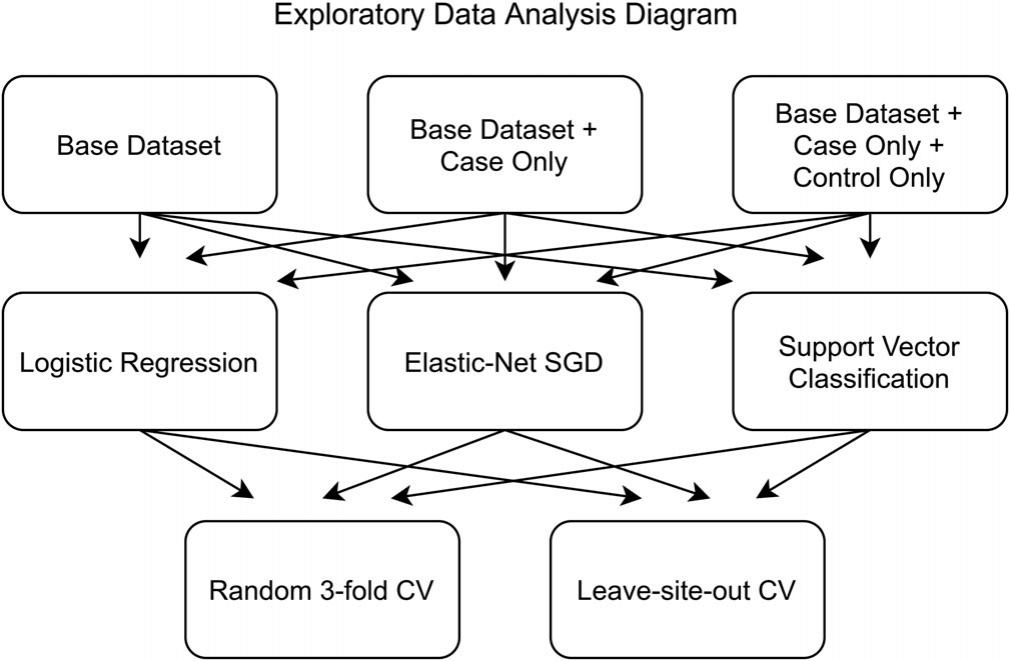
The different permutations of analyses conducted internally on the training set, with differing input dataset options (top row), classifiers (middle row), and computed CV scoring metrics (bottom row)

We explored classifier performance first on a base training dataset (Figure 1, Sites 1–5), composed of the five sites containing a balance of both case and control participants. The same experimental evaluation was then repeated with two augmented versions of the dataset, first adding in participants from case‐only sites (Figure 1, Sites 6–8), and then adding further additional participants from 16 control‐only sites (Figure 1, Sites 9–24). The top row of Figure 2 outlines these three combinations within the context of our experimental design.

Three machine learning algorithms suitable for binary classification (Figure 2, middle row) were implemented within the python library Scikit‐learn (Pedregosa et al., 2011). Feature normalization was conducted in all cases with Scikit‐learn's StandardScaler. Most simply, we considered a regularized ridge logistic regression classifier (l2 loss) with regularization parameter values chosen through an internal CV. Another variant of regularized logistic regression optimized with stochastic gradient descent (SGD) was implemented with an elastic net loss (l1 and l2). A nested random parameter search was conducted, across 100 values, determining the choice of loss function and regularization values (Zou & Hastie, 2005). Finally, we considered a SVM with radial basis function (rbf) kernel, which allowed the classifier to learn nonlinear interactions between features (Suykens & Vandewalle, 1999). Similar to the hyperparameter optimization strategy employed for the SGD logistic regression, a random search over 100 SVM parameter combinations, with differing values for the regularization and kernel coefficients, was employed with nested CV for parameter selection. Exact parameter distributions and training details are provided within the supplemental materials.

Proper CV is of the utmost importance in machine learning applications. It is well known that—if improperly cross‐validated—classifiers can overfit onto validation sets, and even with more sophisticated CV techniques can overestimate expected generalization (Santos, Soares, Abreu, Araujo, & Santos, 2018). Within this work, we employed a random 50 repeated three‐fold CV stratified on AD status, where an indication of classifier performance is given by its averaged performance when trained on one portion of the data and tested on a left‐out portion, across different random partitions (Burman, 1989). We also made use of a leave‐site‐out (or leave‐one‐site‐out) CV scheme across the five sites that include both cases and controls (see Figure 1). Performance for this leave‐site‐out CV is computed as the average score from each site when that site is left out, this is, the average of 5 scores. These options are shown in the bottom row in Figure 2. We computed metrics according to both schemes for all considered classifiers on the training dataset. The area under the Receiver Operating Characteristic curve (AUC) was used as a base performance metric insensitive to class imbalance (DeLong, DeLong, & Clarke‐Pearson, 1988).

### 2.3 Final analytic pipeline

Based on the intermediate results from the previous Exploratory Data Analysis, we implemented a GA designed to select sets of features most useful in training a site generalizable classifier. We operate under the assumption in this stage that if a classifier can be restricted to only features relevant to distinguishing AD versus control, and explicitly not those useful in exploiting site effects, we can create a more robust and generalizable classifier. Toward this goal, the GA repeatedly trained and evaluated a regularized logistic regression classifier on initially random subsets of brain features. The regularized logistic classifier is chosen here as it is quick to train, and the initial exploratory analysis revealed little difference in performance between different classifiers. These feature subsets were then optimized for high AUC scores as determined by the leave‐site‐out CV on the five sites that included both cases and controls. Multi‐objective optimization was conducted with the aid of a number of successful GA strategies, and these include: random tournament selection (Eremeev, 2018), feature set mutations, repeated runs with isolated populations, a sparsity objective similar in function to “Age‐fitness Pareto optimization” (Schmidt & Lipson, 2011), among others. An introduction to GA and a complete description of our design decisions regarding the algorithm are provided in the supplemental material.

**FIGURE 3:**
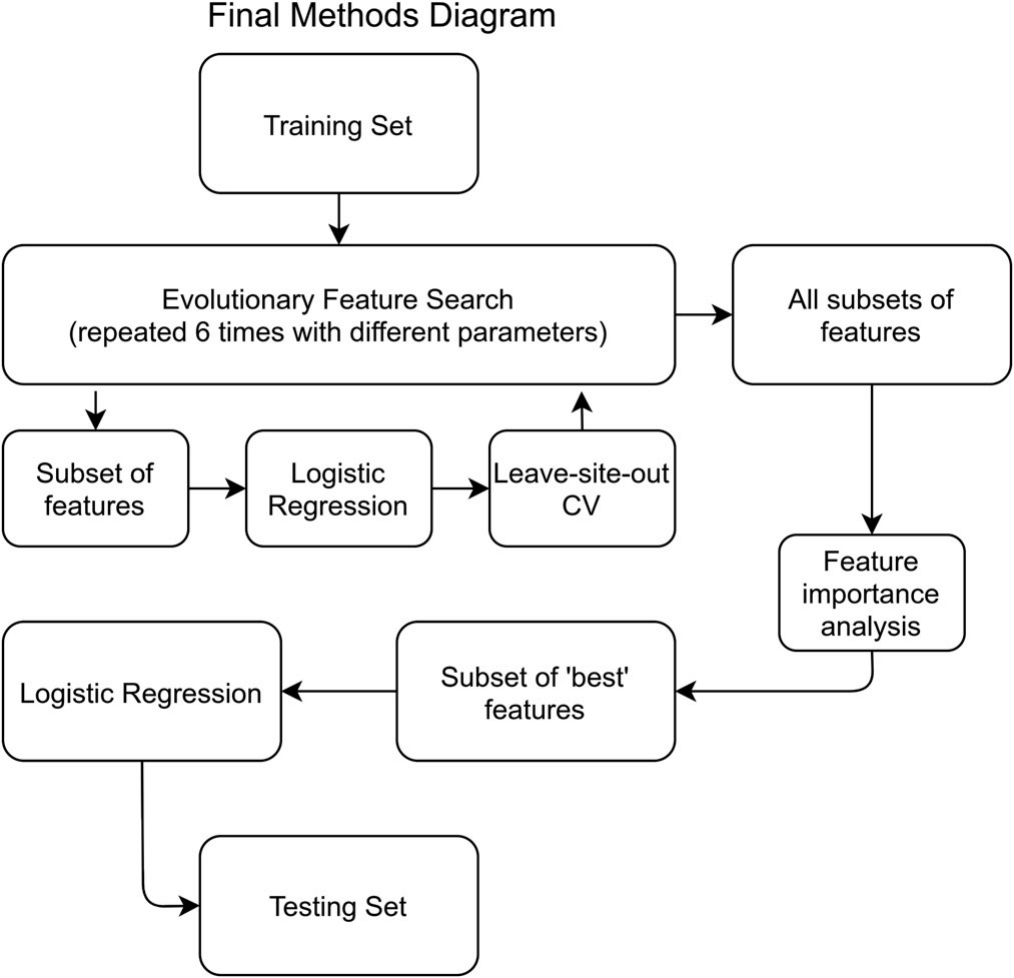
A simplified view of the final pipeline, where the full training dataset is employed in an evolutionary feature search designed to produce optimal subsets of high performing features. From this collection of feature subsets a meta analysis for determining feature importance is conducted and a subset of “best” features are selected. Next, a logistic regression classifier is trained and evaluated on the testing dataset, with access to only the “best” subset of features

The algorithm was run across six different variants of hyperparameters, as shown in Figure 3. We explored choices related to size (number of subsets of features considered in each round) and scope (how many optimization rounds the search is run for) in addition to objective functions. The results from each of the six search variants represent thousands (exact number dependent on hyper‐parameters of that variant) of subsets of features, each with an associated performance score. We restricted the output from each search to the top 200—and therefore to high performing—feature subsets. All of these final feature subsets (1,200 total) were ultimately pooled together and considered in a feature importance meta‐analysis. In determining feature importance, the following considerations were used: each subset's individual performance (higher performance weighted higher) and the number of features (subsets with more features were penalized). A final measure of feature importance was calculated as the average feature importance from each of the six search variants computed separately. Within each search variant, an individual feature's importance was defined as the sum of a feature set's fitness scores, further divided by the number of total features in that set, across all of the top 200 sets in which that feature appeared. Importances per set were then normalized, such that intuitively a feature present in all of the top 200 feature sets would have a value of 1, and if present in none, 0. Each feature's final score represents that feature's averaged score (between 0 and 1) as derived from each separate search. We were interested at this stage in identifying a relative ranking of brain features, as, intuitively, some features should be more helpful in classifying AD, and features that are useful toward classification are candidates to be related to the underlying AD neurobiology.

As referenced in Figure 3, we selected a “best” subset of features with which to train and evaluate a final regularized logistic regression classifier on the withheld testing set. We determined the “best” subset of features to be those which obtained a final feature importance score above a user‐defined threshold. Ideally, this threshold would be determined analytically on an additional independent validation sample, but with limited access to data from case–control balanced sites we employed only internal CV. Posthoc analyses were conducted with differing thresholds, providing an estimate as to how important this step may prove in future analyses.

## 3 RESULTS

### 3.1 Exploratory data analysis

The complete exploratory training set results are shown in Table 2. The base dataset composed of only the five balanced sites across classifiers obtained an AUC of 0.723 to 0.724 under three‐fold CV versus 0.623–0.663 under leave‐site‐out CV. Regularized logistic regression on the base dataset with the addition of extra case‐only subjects yielded an AUC of 0.907 ± 0.022 (standard deviation across folds) under random three‐fold CV versus 0.560 ± 0.189 under leave‐site‐out and with added controls an AUC of 0.917 ± 0.010 with random three‐fold CV and 0.636 ± 0.169 with leave‐site‐out. The choice of classifier produced only minor differences in performance (±.02), regardless of the CV method. The full dataset (including additional control participants and case‐only participants) yielded a small boost to random three‐fold CV scores (.003–.023), and a more noticeable gain to leave‐site‐out CV scores (.053–.091). The CV strategy (Random vs. Leave‐site‐out) produced the largest discrepancy in scores when either case‐only or both case‐only and control‐only participants were included (.267–.347) with the former yielding inflated results.

**TABLE 2:** The results for each of the three considered classifiers with just the base dataset, the base dataset with added case‐only sites and lastly the full dataset with control‐only sites (see Figure 1 for information on which sites are balanced vs. control or case‐only) across both cross validation (CV) strategies, as highlighted in Figure 2

Dataset	Classifier	Random three‐fold CV AUC (± STD)	Leave‐site‐out CV (5 sites) AUC (± STD)
Base	Logistic regression	0.723 ± 0.042	0.644 ± 0.125
Base	SGD	0.731 ± 0.034	0.663 ± 0.139
Base	SVM	0.724 ± 0.038	0.623 ± 0.096
Base ± case‐only	Logistic regression	0.907 ± 0.022	0.560 ± 0.189
Base ± case‐only	SGD	0.896 ± 0.012	0.561 ± 0.183
Base ± case‐only	SVM	0.912 ± 0.011	0.578 ± 0.111
Full (case ± control)	Logistic regression	0.917 ± 0.012	0.636 ± 0.169
Full (case ± control)	SGD	0.919 ± 0.009	0.652 ± 0.132
Full (case ± control)	SVM	0.915 ± 0.014	0.631 ± 0.139

*Note*: Standard deviation in area under the receiver characteristic operator curve (AUC) across cross‐validated folds is provided, as an estimate of confidence. Random three‐fold CV was stratified according to AD status and was repeated 50 times with different random splits.

### 3.2 Feature importance

**FIGURE 4:**
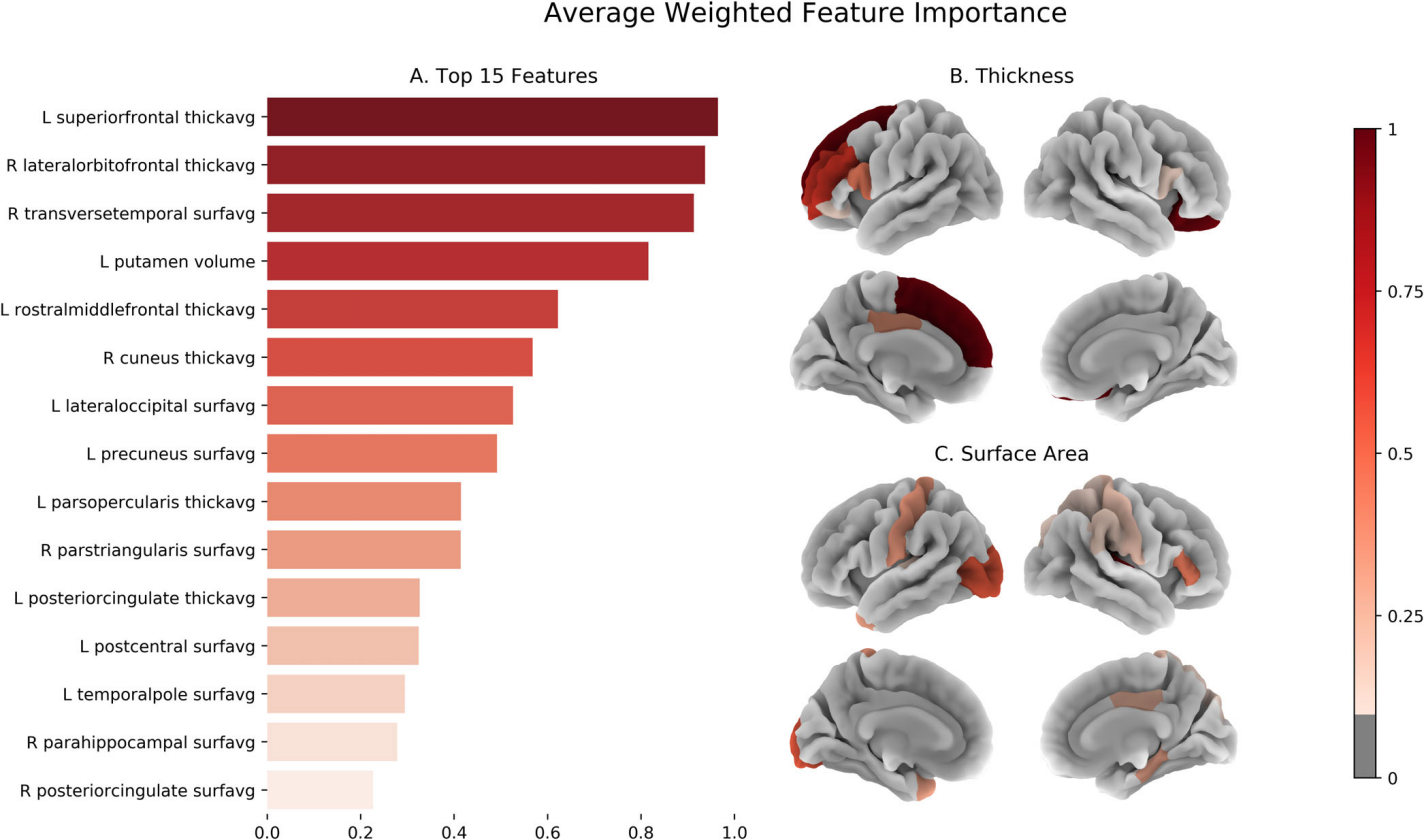
(a) The top 15 features (threshold chosen for readability), as ranked by average weighted feature importance (where 0 indicates a feature appeared in none of the GA final models, and 1 represents a feature appeared in all) are shown. (b) The cortical thickness and (c) cortical average surface area feature importance scores, above an a priori selected threshold of 0.1, are shown as projected onto the fsaverage surface space

The top 15 features as determined by average weighted feature importance, from all six searches (i.e., base training dataset only and base plus control‐only datasets, by three machine‐learning algorithms; see Figure 2), are presented in Figure 4. Four features emerged with an importance score greater than 0.8 (where an importance score of 1 represents a feature present in every top feature set and 0 in none), followed by a slightly sharper decline and, not shown, a continuing decline. Also shown are the cortical surface area and thickness features as projected onto the *fsaverage* cortical surface space. The left putamen (0.816) and left pallidum (0.210) volumes were the only subcortical features with feature importance scores over 0.05 (not shown).

### 3.3 Testing set evaluation

**FIGURE 5:**
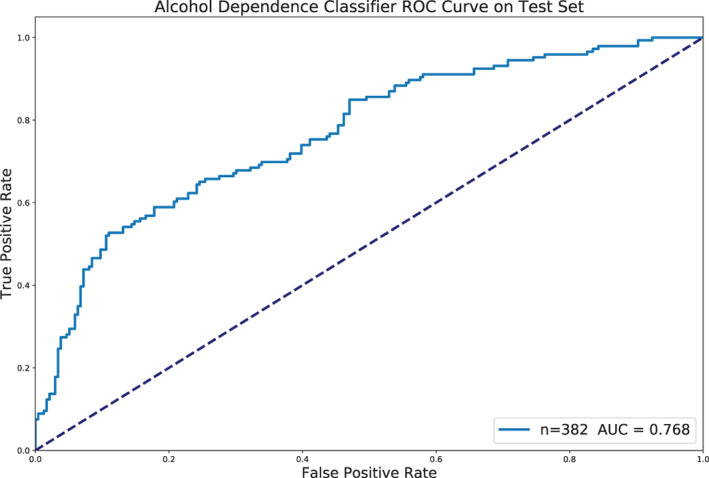
The ROC curve for the final logistic regression model on the testing set, as restricted to only the “best” subset of four features

Further internal nested validation on the training set selected a threshold of 0.8 weighted feature importance and above, which corresponds to the top four features only (Figure 4). The final model, trained on only this “best” subset of four features, achieved an AUC of 0.768 on the testing set. The ROC curve for this classifier on the testing set is shown in Figure 5. We further conducted a number of posthoc analyses on the testing dataset. To confirm the predictive utility of GA feature selection, a regularized logistic regression model and SVM model with access to all features were both trained on the full training dataset and evaluated on the testing set. The logistic regression scored 0.697 AUC and the SVM 0.673 AUC. Similarly, regularized logistic regression and SVM models were trained on all features, but without the inclusion of additional control‐only sites, and scored, respectively 0.647 and 0.609 AUC. The final model was better than both the logistic regression model with all features and subjects (*p* = .0098) and without control subjects (*p* = 3.5 × 10^−5^). We then trained on just the five balanced sites, where logistic regression scored 0.717 AUC and the SVM 0.700 AUC. We further investigated the choice of user‐defined threshold in selecting the number of top features by testing the inclusion of the top 2 to 15 features. Some notable differences can be seen in performance, for example: .782 AUC with top three, .737 AUC with top five and 0.741 AUC with top 10.

## 4 DISCUSSION

We used multi‐site neuroimaging data to identify structural brain features that classify new participants, from new sites, as having an AD with high accuracy. In doing so, we highlighted the importance of carefully chosen metrics in accurately estimating ML classifier performance in the context of multi‐site imbalanced neuroimaging datasets. We further explored a number of techniques, ranging from analytical methods to more general approaches, and their merit toward improving classifier performance and generalizability. Our proposed GA‐derived feature importance measure, in addition to aiding classification, might help in identifying neurobiologically meaningful effects.

A clear discrepancy arose between random repeated CV (i.e., participants randomly selected from across sites) and leave‐site‐out CV results (Table 2). We suspect that the random repeated CV overestimates performance due to covert site effects. The classifiers appeared to memorize some set of attributes, unrelated to AD, within the case‐ and control‐only sites and therefore were able to accurately predict AD only if participants from a given site were present in both training and validation folds. This is exemplified by the change in performance seen when case‐only subjects are added, where repeated three‐fold CV goes up ~0.18 AUC, but leave‐site‐out CV drops ~0.08 AUC. Performance on leave‐site‐out CV, in contrast to random repeated CV, better estimates classifier generalizability to new unseen sites, especially when the dataset contains data from any case‐only or control‐only sites. This is validated by post hoc analyses in which a logistic regression trained on all features obtained a test set AUC (0.697) far closer to its training set leave‐site‐out CV score (0.636 ± .119) then its random repeated CV score on the full training set (0.917). While this observation must be interpreted within the scope of our presented imbalanced dataset, these results stress the importance of choosing an appropriate performance metric, and further highlight the magnitude of error that can be introduced when this metric is chosen incorrectly.

In addition to performing model and parameter selection based on a more accurate internal metric, the addition of control‐only participants relative to when just case‐only subjects are included proved beneficial to classifier performance (0.053–0.091 gain in leave‐site‐out AUC). This effect can be noted within our exploratory data analysis results (Table 2) comparing leave‐site‐out CV results between the base dataset plus case‐only subjects and the full dataset. When extra control participants are added performance increased up to 0.09 AUC. Posthoc analysis revealed a similar performance gain on the testing set from adding control participants; logistic regression plus 0.05 AUC and SVM plus 0.06 AUC. This boost likely reflects a combination of two circumstances. In the first, the underlying ML algorithm is aided by both more data points to learn from and a more balanced case to control distribution, which have both been shown to aid binary classification performance (Jordan & Mitchell, 2015). The second reflects a resistance to the learning of site‐related effects which, as noted above, can lead to the algorithm detrimentally learning covert site effects. By including data from more sites and scanners, it is possible the unintentional learning of specific site effects (as a proxy for AD) is made more difficult. More generally, as neuroimaging data banks continue to grow, the potential arises for supplementing analyses with seemingly unrelated external datasets.

Between‐site variance, leading ML classifiers to exploit trivial site differences, is a pernicious, but not wholly unexpected problem. One source of this variance is likely related to scanning differences, that is, manufacturer, acquisition parameters, field inhomogeneities and other well‐known differences (Chen et al., 2014; Jovicich et al., 2006; Martinez‐Murcia et al., 2017). Data pooled from studies around the world also introduce sociodemographic differences between sites. Importantly, the clinical measure of interest is also often variable (see Supporting Information for more information on the different diagnostic instruments used in our sample) (Yip et al., 2020). Especially when pooling studies, it is difficult to fully control or correct for all of these sources of variances, as different studies will use a range of different scanning protocols and collect nonoverlapping phenotypic measures. Despite a potential host of differences, pooled data from multiple sites may actually provide a regularizing effect. For example, if only a single diagnostic instrument were employed a classifier may obtain strong within‐study results, but be unlikely to generalize well to new studies utilizing alternative instruments.

Our proposed GA‐based feature selection, with the inclusion of leave‐site‐out criteria, proved to be useful in improving classifier generalizability. This is highlighted by a 0.071 boost to AUC score in a model trained on only the top identified four features in contrast to a model trained with all the available features. We believe the observed performance boost to be a result of only allowing the classifier to learn from features previously determined to be useful toward site generalizable classification. In this way, the final classifier is able to avoid adverse site effects through a lack of exposure to brain measurements highly linked to specific sites. We note also that our final proposed classifier compares favorably to the other posthoc comparisons conducted. Specifically, we see a 0.095 boost relative to an SVM trained on the full dataset, a 0.121 and 0.159 boost relative to regularized logistic regression and SVM models trained on the base dataset with added cases (or full dataset minus extra controls), and lastly a 0.051 and 0.068 gain relative to just the base dataset. Further posthoc results indicate even higher performance with just the top three features (+.014 vs. selected top four feature model) and a slight decrease with the addition of more features. In future work, an additional validation set might prove useful in selecting between different final models and thresholds, in addition to careful comparisons between different feature selection methods.

A persistent issue in typical interpretation from ML models is the issue of shared variance between different features. The features a single model selects may very well have suitable surrogate features within the remaining dataset. In contrast, our feature importance metric is derived from thousands of models, providing the chance for equivalent features, with shared variance, to achieve similar importance scores. A natural distinction nevertheless exists between predictive features and those that emerge from univariate testing as significant. Specifically, the absence of a feature within our final model, (i.e., the unimportance of a feature by our metric), does not necessarily imply a lack of association between that feature and AD. An absence could alternatively indicate that a different feature better captures some overlapping predictive utility, which is different conceptually from sharing variance in that in this case one feature is consistently more useful for prediction. The redundant feature might not appear as important despite an association with AD when considered in isolation. On the other hand, a feature with a relatively weak association could emerge with consistently high feature importance if it proves uniquely beneficial to prediction. Above and beyond univariate significance, if a given feature does have predictive utility, it strongly suggests that a real association exists. Our selected top features were both identified as consistently useful features within the training set and experimentally confirmed as site generalizable on the testing set.

The top four features as identified by our metric of feature importance were the average cortical thickness of the left superior frontal gyrus and right lateral orbitofrontal cortex (OFC), the left putamen volume and the average surface area of the right transverse temporal gyrus. Specifically, cortical thinning, volume and surface area reduction across these regions prompt the trained model toward an AD prediction. Thinning, within the left superior frontal gyrus and right lateral OFC, agrees broadly with the literature which has consistently shown frontal lobe regions to be most vulnerable to alcohol consequences (Oscar‐Berman & Marinković, 2007). Prefrontal cortical thinning and reduced volume in the left putamen seem to further indicate specific involvement of the mesocorticolimbic system. This dopaminergic brain pathway has been consistently linked with alcohol dependence and addiction in general (Ewing et al., 2014; Filbey et al., 2008). Likewise, a recent voxel‐based meta‐analysis showed a significant association between lifetime alcohol consumption and decreased volume in left putamen and left middle frontal gyrus (Yang et al., 2016).

Comparing the four selected regions in the present study with those determined to be significant by univariate testing on an overlapping dataset from Mackey et al., 2019, we find three regions in common (the exception being right transverse temporal gyrus surface, as surface area was not considered in that analysis). Further, left superior frontal and putamen appeared as two of the top 20 features in both folds of an SVM classifier trained and tested on split halves in the Mackey paper (right lateral orbital frontal only appeared in one fold). Of the existing alcohol classifiers mentioned in the introduction by Guggenmos et al. (2018) and Adeli, Zahr, Pfefferbaum, Sullivan, and Pohl (2019), only Adeli reported overlapping AD‐associated regions with our top four: lateral orbitofrontal thickness and superior frontal volume.

In interpreting the performance of a classifier linking brain measurements to an external phenotype of interest, we also need to consider how reliably the phenotype can be measured. The exact relationship between interobserver variability of a phenotype or specific diagnosis and ease of predictability or upper bound of predictability is unknown, but it seems plausible that they would be related. This proves pertinent in any case where the presented ground truth labels, those used to generate performance metrics, are noisy. We believe further study quantifying these relationships will be an important next step toward interpreting the results of neuroimaging‐based classification, as even if a classifier capable of perfectly predicting between case and control existed, it would be bound by our current diagnostic standard. A potential route toward establishing a robust understanding of brain changes associated with AD might involve some combination of standard diagnostic practices with objective measures or indices gleaned from brain‐based classifiers. Relating classifiers directly with specific treatment outcomes (potential index for recovery), or within a longitudinal screening context (potential index for risk) represent further exciting and useful applications.

We have drawn attention to the impact on model generalizability of case distribution by site within large multi‐site neuroimaging studies. In particular, we have shown that CV methods that do not properly account for site can drastically overestimate results, and presented a leave‐site‐out CV scheme as a better framework to estimate model generalization. We further presented an evolutionary‐based feature selection method aimed at extracting usable information from case‐ and control‐only sites, and showed how this method can produce more interpretable, generalizable and high‐performing AD classifiers. Finally, a measure of feature importance was used to determine relevant predictive features, and we discussed their potential contribution to our understanding of AD neurobiology.

## Supporting information


**Appendix** S1: Supporting Information.

## Data Availability

Data Availability Statement: Data was gathered by the Enigma Addiction Consortium (https://www.enigmaaddictionconsortium.com/). Sharing data publically is not possible due to privacy concerns around protected information, but interested researchers should contact the Enigma Addiction Consortium for more information at enigma.addiction.consortium@gmail.com. Code Availability Statement: Code used in the evolutionary search, along with most postanalysis code is provided at https://github.com/sahahn/Alc_Dep. Note: If anyone is interested in directly replicating the methods used they should contact sahahn@uvm.edu, who if there is interest is willing to provide a more user friendly (and less bound to running on a cluster) version of the methods used.
