# Supplementary material for: Predicting alcohol dependence from multi‐site brain structural measures
Source: Hum Brain Mapp. 2020 Oct 16;43(1):555–65. doi: 10.1002/hbm.25248 (PMC8675424; doi:10.1002/hbm.25248)
Supplement: Supplementary file 1 — Appendix S1: Supporting Information. [file HBM-43-555-s001.zip › HBM_25248_Data and Code Availability (2).pdf]

Data Availability Statement:
Data was gathered by the Enigma Addiction Consortium (https://www.enigmaaddictionconsortium.com/). Sharing data publically is not possible due to privacy concerns around protected information, but interested researchers should contact the Enigma Addiction Consortium for more information at enigma.addiction.consortium@gmail.com.

Code Availability Statement:
Code used in the evolutionary search, along with most post analysis code is provided at https://github.com/sahahn/Alc_Dep. Note: If anyone is interested in directly replicating the methods used they should contact sahahn@uvm.edu, who if there is interest is willing to provide a more user friendly (and less bound to running on a cluster) version of the methods used.
