# Supplementary material for: Predicting alcohol dependence from multi‐site brain structural measures
Source: Hum Brain Mapp. 2020 Oct 16;43(1):555–65. doi: 10.1002/hbm.25248 (PMC8675424; doi:10.1002/hbm.25248)
Supplement: Supplementary file 1 — Appendix S1: Supporting Information. [file HBM-43-555-s001.zip › HBM_25248_Supplemental Material.docx]

**Supplemental Material**

**Site information**

Additional information on the majority of studies/sites can be found in the supplemental materials of <https://www.ncbi.nlm.nih.gov/pmc/articles/PMC6427822/> (<https://www.ncbi.nlm.nih.gov/pmc/articles/PMC6427822/bin/NIHMS1002848-supplement-1.pdf>). The following table lists a conversion between internal site number as references within Figure 1 and the text, and PI / Study Name as listed within the resource linked above. Only sites described previously are listed here, data from new sites are described below the following table.

| Study #  (as referenced in Figure 1) | PI | Study Name | Participants dropped for any missing ROI data |
| --- | --- | --- | --- |
| 1 | Hutchison | ETOH | 3 |
| 2 | Hutchison | Olanzapine | 9 |
| 3 | Sinha | IRC | 34 |
| 4 | Momenan | NIAAA | 117 |
| 5 | Wiers | NeuroADAPT | 17 |
| 6 | Schmaal/ Veltman | TrIP study | 9 |
| 7 | Sjoerds/ Veltman | NESDA-AD | 50 |
| 8 | Goudriaan / Van Holst | ADPG study | 0 |
| 9 | London | Young Smokers | 11 |
| 10 | E Stein | Smokers | 3 |
| 11 | Luijten/ Veltman | DABIS | 5 |
| 12 | Garavan/ Foxe |  | 1 |
| 13 | Sinha | SCOR | 10 |
| 14 | E Stein |  | 5 |
| 15 | London |  | 15 |
| 16 | Paulus | Relapse | 2 |
| 17 | D Stein | Meth-CT | 26 |
| 18 | Garavan/ Hester | Trinity-THC | 0 |
| 19 | Garavan | Orr | 0 |
| 20 | Cousijn/ Goudriaan | Cannabis Prospective | 0 |
| 21 | Allen | ADS | 26 |
| 22 | Martin - Santos | Chronic cannabis users (Barcelona) | 12 |
| 23 | Solowij | Chronic Cannabis | 6 |
| 24 | Yucel | Chronic Cannabis - Memory | 13 |

New data not included in “Mega-Analysis of Gray Matter Volume in Substance Dependence: General and Substance-Specific Regional Effects”, spans internally labelled sites 25, 26 and 27. These sites are from the NFGNplus study by PI Henrik Walter. Alcohol Dependent participant inclusion criteria were diagnosis of alcohol dependence [Diagnostic and Statistical Manual of Mental Disorders, Fourth Edition (DSM‐IV), Structured Clinical Interview for DSM‐IV Axis I Disorders (SCID‐I); First et al. 2001] and completion of medically supervised detoxification.

Structural scans were also acquired using a T1-weighted three-dimensional MP-RAGE sequence with an isotropic spatial resolution of 1 mm3. At Charité Berlin, ten subjects were scanned using a Siemens Trio 3T MR and 45 subjects were scanned using a MAGNETOM Verio 3T (Siemens, Erlangen, Germany) using the same acquisition parameters (TR=2.3s, TE=3.03ms, flip angle = 9°). At the Life and Brain Center of the University of Bonn, subjects were scanned using a Siemens Trio 3T MR (TR=2.3s, TE=3.93ms, flip angle = 9°). At the Central Institute of Mental Health Mannheim, subjects were scanned using a Siemens Trio 3T MR (TR = 2.3s, TE = 3.03ms, flip angle = 9°).

**Exploratory Data Analysis classifier details**

Each of the three classifiers trained and evaluated during the exploratory section were implemented through scikit-learn. We performed feature normalization using Scitkit-learn’s StandardScaler on the training set as a whole, and on the testing set separately. In hindsight, this normalization should have been conducted on the fly, for example, in the context of a Scikit-learn Pipeline, but we feel in this case the effect should be very negligible (and is therefore not worth repeating all experiments). To confirm this intuition, we re-ran the analysis mentioned in the Exploratory Data Analysis section with properly nested scaling and achieved nearly identical results (+- .001). Across all three classifiers, the ‘class_weight’ parameter was set to ‘balanced’, where internal weights are adjusted according to input class frequencies (ratio of case to control), potentially helping handle class imbalance. The stochastic gradient descent (SGD) and support vector machine (SVM) implementations were further wrapped in scikit-learn’s RandomizedSearchCV object in order to select hyperparameters through random search with nested 5-fold cross validation. 100 random parameters were tested when optimizing the respective classifier. Exact parameters, and parameter distributions explored are listed below.

Logistic Regression:

The logistic classifier was the scikit-learn class LogisticRegressionCV. This classifier incorporates 5-fold cross validation into selection of hyperparameter C (the inverse of regularization strength). It specifically considers 10 C values on a logarithmic scale between 1e-4 and 1e4. The regularization strength refers to the scaling applied to l2 regularization, also known as ridge regression. We made use of the ‘lbfgs’ (Limited memory Broyden–Fletcher–Goldfarb–Shanno) solver, which is the optimization algorithm used.

SGD:

We utilized the scikit-learn class SGDClassifier. We specified that an elastic-net penalty be enforced, which combines l1 and l2 regularization though the ‘l1_ratio’ hyperparameter (values between 0 and 1, with 1 representing just l1 loss). Random values between 0 and 1 were provided to the random parameter search, along with random values logarithmically distributed between 1e-4 and 1e3 for the choice of ‘alpha’ parameter (which defines the regularization strength). Lastly, the choice between two loss functions was provided as a hyperparameter, specifically between a ‘modified huber’ (smooth loss with tolerance to outliers) and ‘log’ loss (equivalent to logistic regression).

SVM:

We utilized the scikit-learn class SVC (support vector classifier). The ‘rbf’ (radial basis function) kernel was utilized, which allows the SVM to learn non-linear interactions, but greatly increased complexity. The parameters ‘C’ and ‘gamma’ were selected through random hyperparameter search. ‘C’ refers to the inverse regularization strength and was provided with random logarithmically distributed choices between 1 and 1e5. ‘gamma’ refers to the kernel coefficient and was provided with random logarithmically distributed choices between 1e-5 and 1e3.

Scikit-learn documentation links:

-<https://scikit-learn.org/stable/modules/generated/sklearn.model_selection.RandomizedSearchCV.html>

-<https://scikit-learn.org/stable/modules/generated/sklearn.linear_model.LogisticRegressionCV.html>

-<https://scikit-learn.org/stable/modules/generated/sklearn.linear_model.SGDClassifier.html>

-<https://scikit-learn.org/stable/modules/generated/sklearn.svm.SVC.html>

**Base Genetic Algorithm**

A single GA search is a population-based approach designed roughly to mimic evolutionary principles. First, we initialize a fixed number of populations, where each population is composed of another fixed number of individuals. Each individual represents a subset of brain features, with 3-5 randomly selected upon initialization. After all individuals in the population have been initialized, a given number of evolutionary generations are run. The number of populations, individuals within a population, and number of generations for which each population is run, all represent search hyper-parameters which we address with the different size variants (described below).

Each evolutionary generation is made up of an evaluation, removal and replacement phase. First, all individuals within each population are evaluated by a fitness function and assigned a fitness score accordingly. We define a given individual’s fitness score as the ROC obtained from running a leave-out site CV with a regularized logistic classifier with access to only that individual’s subset of brain features. Low performing individuals are then removed using a strategy known as random tournament selection. This selection procedure continually compares two randomly selected individuals from the population and removes an individual only if dominated by its selected counterpart. This procedure is repeated until exactly half of the population remains. Within our multi-objective optimization formulation, we defined an individual as dominating another if and only if that individual is better than the other according to both objective functions (described below). Lastly, the removed individuals are replaced with mutated copies randomly chosen from the remaining individuals as well as one newly initialized individual (adding new genetic diversity to the population). We defined our mutation strategy as first creating a copy of an existing individual, and then with a fixed probability adding (24%), changing (60%) or removing (16%) a feature from the new individual’s subset of brain features. Probabilities were chosen as “reasonable” values, in order to lower the global number of hyper-parameters.

**Genetic Algorithm Objectives and Search Parameters**

GA for feature selection are known to overfit easily onto the question of interest, and can likewise produce different results from run to run or when given different hyper-parameters. Due to this inherent overfitting and instability, we ran six unique searches in order to explore a broad range of possible solutions. The underlying idea is that while a single search on its own might have a suboptimal fitness function or be under or over-fit onto the performance metric, by averaging over a diverse range of searches we can obtain robust results. We implemented three different pairs of GA objectives, an objective typically representing a fitness function or penalization. Each pair of objectives constitutes its own multi-objective GA search, and each pair of objectives is used twice in both a ‘small’ and ‘large’ variant. These objectives and size variants are described in detail below.

We limited the number of objectives within a single GA search to two, as with each additional objective dimension, the size of the Pareto front of individuals grows exponentially (Van, et al 1998). On the other hand, by using two objectives at once, we are able to retain an expressive and exploratory population. A Pareto front refers to the set of individuals that cannot be dominated, as mentioned above, by any other individual within the population. The Pareto front of individuals at the final evolutionary generation, therefore, constitutes the set of approximate solutions to the provided multiple objectives. Within all pairs of objectives, we included at least one representation of fitness score (an individual's ROC score as computed from leave-out site CV). As this score was determined from five different sites, all with a differing number of participants (Figure 1 sites 3,4,5,6,8), we computed a ROC score disregarding site (Micro ROC), an unweighted mean from the ROC score obtained on each site separately (Macro ROC), and the minimum ROC score from any one site (Min ROC). A summary fitness score (Summary Fitness) is further calculated as ¼ Macro ROC + ¼ Micro ROC + ½ Min ROC. These unique fitness scores (and weightings) were selected in order to obtain a diverse array of solutions. In two of the three pairs of objectives, we employed a sparsity constraint (Sparsity) as our second objective in addition to fitness score. Our sparsity constraint dictated that an individual with fewer features could not be dominated within the tournament selection except by another individual with equal to or less than the same number of features. When combined with the fitness score objective, both the highest performing individuals, regardless of size, as well as decently performing smaller individuals, can co-exist within the same population, akin to the effective multi-objective optimization strategy described in “Age-fitness Pareto optimization” (Schmidt, et al. 2011).

We applied the following three pairs of objective functions: 1. Macro ROC and Sparsity, 2. Macro ROC and Micro ROC and 3. Summary Fitness and Sparsity. Each pair of objectives was run with a fixed ‘small’ and ‘large’ selection of search parameters producing a total of six unique runs. The ‘small’ variant had 100 individuals per population and was run over 500 generations, whereas the ‘large’ variant had 200 individuals per population and was run over 2000 generations. Both variants employed 50 populations. Altogether, this comprised six sets of unique hyperparameters, as referenced within Figure 3. We employed different search variants (combinations of fitness functions and different sizes) both in order to explore a larger search space of solutions and in an attempt to combat overfitting onto our training set.

**Feature Importance**

We defined a summary measure of feature importance with the goal of leveraging useful information contained across all of the different searches. All searches were not run with the same objectives and therefore could not be compared directly. To address this, we conducted feature importance analyses (as described below) independently on the output from each search. Average feature importance was then established as the mean of all six individual weighted feature importance scores.

Each search generated either 5,000 (50 populations * 100 individuals) or 10,000 (50 populations * 200 individuals) sets of feature subsets (individuals). We limited these feature subsets to only the top 200 as ordered by an individual’s fitness score, where fitness score was determined by that search variant’s fitness function objective (in the case with two fitness functions, macro ROC was used). Next, we defined an importance score for each feature as the sum of the fitness values across each individual in which that feature occurs. This results in a higher weighting for features that occur more often within high performing individuals and allows us to define a relative ordering between features. Scores are then normalized within each search, such that a feature with a score of 1 would indicate that feature appeared in all 200 top subsets. Explicitly, normalization is conducted by dividing all features’ weighted sums with the weighted sum of all individuals.

**Selecting a final subset of Features**

While we recommend in future work that a final top subset of features in which to restrict a final model be determined from an additional validation set of data, within this work we employed additional internal CV on the training set data. Specifically, we evaluated a regularized logistic regression in the same leave-one-site-out manner as in earlier analysis, but this time across all of only the unbalanced (either case only or control only) sites. This process generates predicted scores for each site left out, and ultimately predicted scores for every sample from an unbalanced site. We considered a composite metric as the comparison metric of interest, which we call the “unbalanced score”. We will first introduce the base pieces of the unbalanced score, and then work up to how it is ultimately composed.

We compute a measure of micro and macro accuracy, where micro refers to computing accuracy across all samples at the same time, versus macro which considers first computing accuracy within each site independently and then averaging across each site’s accuracy. In a similar manner, we also computed a measure of micro roc auc (where computing macro roc auc is not possible given each specific site does not have the presence of both cases and controls). We further consider the minimum accuracy obtained on any one site. These base metrics are then combined as follows: an average accuracy is computed as the mean of the micro accuracy and macro accuracy. Then, the mean of the average accuracy, minimum accuracy and micro roc auc are taken to define the final unbalanced score.

The rationale for creating such a complex composite score is that each metric when considered alone has its own flaws. For example, accuracy is not sensitive to imbalances in case to control. Likewise, macro measures are not sensitive to differences in numbers of subjects per site, whereas micro measures are too sensitive to big differences in numbers of subjects per site.

The proposed unbalanced score is then employed only as a way of comparing regularized logistic regression models trained on different subsets of the computed top features. We compute this score for all possibilities for the number of top features to include, e.g., just the top 3, 4, 5, 6, etc… and in this way selected ultimately the inclusion of just the top 4 features.

**Code Availability Statement**

Code used in the evolutionary search, along with most post analysis code is provided at <https://github.com/sahahn/Alc_Dep>. If any interested parties are directly interested in replicating the methods used, they should contact [sahahn@uvm.edu](mailto:sahahn@uvm.edu) directly.

**Data Availability Statement**

Data was gathered by the Enigma Addiction Consortium (<https://www.enigmaaddictionconsortium.com/>). Sharing data publically is not possible due to privacy concerns around protected information, but interested researchers should contact the Enigma Addiction Consortium for more information at ENIGMA.addiction@uvm.edu.

.
